# Supplementary material for: CiFi: accurate long-read chromosome conformation capture with low-input requirements
Source: Nat Commun. 2025 Dec 8;17:215. doi: 10.1038/s41467-025-66918-y (PMC12780124; doi:10.1038/s41467-025-66918-y)
Supplement: Supplementary file 2 — Description of Additional Supplementary Information [file 41467_2025_66918_MOESM2_ESM.pdf]

Description of Additional Supplementary Files

File Name: Supplementary Data 1
Description: PacBio sequencing statistics.

File Name: Supplementary Data 2
Description: CiFi mapping segment summary statistics.

File Name: Supplementary Data 3
Description: CiFi protocol modifications for reduced lymphoblastoid cell line input.
