# Supplementary material for: CiFi: accurate long-read chromosome conformation capture with low-input requirements
Source: Nat Commun. 2025 Dec 8;17:215. doi: 10.1038/s41467-025-66918-y (PMC12780124; doi:10.1038/s41467-025-66918-y)
Supplement: Supplementary file 6 — Reporting Summary [file 41467_2025_66918_MOESM6_ESM.pdf]

nature portfolio

Corresponding author(s): Megan Y. Dennis, Jonas Korlach

Last updated by author(s): Sep 4, 2025

Reporting Summary

Nature Portfolio wishes to improve the reproducibility of the work that we publish. This form provides structure for consistency and transparency in reporting. For further information on Nature Portfolio policies, see our Editorial Policies and the Editorial Policy Checklist.

Statistics

For all statistical analyses, confirm that the following items are present in the figure legend, table legend, main text, or Methods section.

n/a	Confirmed	
[ ]	[x]	The exact sample size (*n*) for each experimental group/condition, given as a discrete number and unit of measurement
[x]	[ ]	A statement on whether measurements were taken from distinct samples or whether the same sample was measured repeatedly
[x]	[ ]	The statistical test(s) used AND whether they are one- or two-sided<br>*Only common tests should be described solely by name; describe more complex techniques in the Methods section.*
[x]	[ ]	A description of all covariates tested
[x]	[ ]	A description of any assumptions or corrections, such as tests of normality and adjustment for multiple comparisons
[ ]	[x]	A full description of the statistical parameters including central tendency (e.g. means) or other basic estimates (e.g. regression coefficient) AND variation (e.g. standard deviation) or associated estimates of uncertainty (e.g. confidence intervals)
[x]	[ ]	For null hypothesis testing, the test statistic (e.g. *F*, *t*, *r*) with confidence intervals, effect sizes, degrees of freedom and *P* value noted<br>*Give P values as exact values whenever suitable.*
[x]	[ ]	For Bayesian analysis, information on the choice of priors and Markov chain Monte Carlo settings
[x]	[ ]	For hierarchical and complex designs, identification of the appropriate level for tests and full reporting of outcomes
[ ]	[x]	Estimates of effect sizes (e.g. Cohen's *d*, Pearson's *r*), indicating how they were calculated

Our web collection on statistics for biologists contains articles on many of the points above.

Software and code

Policy information about availability of computer code

Data collection

Nextflow v23.04.3 Workflow system for creating scalable, portable, and reproducible workflows
fastcat = 0.15.1: Software for creating summaries from standard bioinformatics formats
mosdepth = 0.3.8: Software for fast BAM/CRAM depth calculation
pairtools = 1.1.2: Software framework for processing Hi-C data
whatshap = 2.1: Software for phasing genomic variants using DNA sequencing reads
pore-c-py = 2.0.6: Python scripts for working with Pore-C data
samtools = 1.17: Utilities for manipulating high-throughput sequencing data
minimap2 = 2.28 : Sequence alignment program for long-read mapping

Data analysis

bedtools = 2.26.0 : A suite of tools for genomic data analys
htslib = 1.21 : Library for high-throughput sequencing data formats
minimap2 = 2.26 : Sequence alignment program for long-read mapping
mosdepth = 0.3.10 : Software for fast BAM/CRAM depth calculation
samtools = 1.21 : Utilities for manipulating high-throughput sequencing data
juicer_tools = 1.9.9 A set of tools for processing and analyzing Hi-C data
Juicebox = v1.11.08 Visualization software for Hi-C data
r-SVbyEye = 0.99.0: A visual tool to characterize structural variation
r-TopDom = 0.10.1.9003: Software for Identifying Topological Domains in Genomes
r-base = 4.3.3 : R statistical computing environment

r-ggplot2 = 3.5.1 : Data visualization package for R
r-dplyr = 1.1.4 : Data manipulation package for R
r-tidyverse = Various : Collection of R packages for data science
r-data.table= 1.16.4: Package for fast processing of data
r-readr = 2.1.5 : Fast reading of large rectangular data files in R
whatshap = 2.8: Software for phasing genomic variants using DNA sequencing reads
HiFiasm = 0.24.0-r702 Haplotype-resolved de novo assembler
BUSCO = 5.7.1 Pipeline for assessing the quality of prokaryotic and eukaryotic genomes
YAHS = 1.2a.2 Scaffolding tool using Hi-C data
pbmarkdup = 1.1.0 Tool for marking duplicate reads from PacBio sequencing of an amplified library
EarlGrey = 5.1.1 A full-automated transposable element (TE) annotation pipeline
StaiinedGlass = 0.6 interactive visualization of massive tandem repeat structures with identity heatmaps
HiGlass = 1.13 A tool for exploring and compare genomic contact matrices and tracks
BLAST Basic Local Alignment Search Tool
ULTRA = 1.1.0 A tool for finding and annotating tandem repeats within genomic sequence
YAK = 0.1 Yet another k-mer analyzer used for calculating base accuracy of assemblies
BBMap = 35.85 A Fast, Accurate, Splice-Aware Aligner

For manuscripts utilizing custom algorithms or software that are central to the research but not yet described in published literature, software must be made available to editors and reviewers. We strongly encourage code deposition in a community repository (e.g. GitHub). See the Nature Portfolio guidelines for submitting code & software for further information.

Data

Policy information about availability of data

All manuscripts must include a data availability statement. This statement should provide the following information, where applicable:

- Accession codes, unique identifiers, or web links for publicly available datasets
- A description of any restrictions on data availability
- For clinical datasets or third party data, please ensure that the statement adheres to our policy

All data is available through the European Nucleotide Archive and NCBI GenBank through accession PRJEB83708.

Research involving human participants, their data, or biological material

Policy information about studies with human participants or human data. See also policy information about sex, gender (identity/presentation), and sexual orientation and race, ethnicity and racism.

Reporting on sex and gender	While GM12878 lymphoblastoid cell line is derived from a biologically female human. There were no comparisons between individuals.
Reporting on race, ethnicity, or other socially relevant groupings	GM12878 is a CEPH 1000 Genomes Individual of European ancestry. Ancestry is well known and public information for this cell line.
Population characteristics	No population information was used in this study.
Recruitment	No recruitment was performed for this study.
Ethics oversight	This study was reviewed by the UC Davis IRB and found to be Human Subject exempt.

Note that full information on the approval of the study protocol must also be provided in the manuscript.

Field-specific reporting

Please select the one below that is the best fit for your research. If you are not sure, read the appropriate sections before making your selection.

[x] Life sciences   [ ] Behavioural & social sciences   [ ] Ecological, evolutionary & environmental sciences

For a reference copy of the document with all sections, see nature.com/documents/nr-reporting-summary-flat.pdf

Life sciences study design

All studies must disclose on these points even when the disclosure is negative.

Sample size	This methods research study used a sample size of one per species as a proof of principle.
Data exclusions	No data were excluded from the analysis.
Replication	For GM12878, multiple replicates were performed using different cell abundances and different restriction enzymes. For the Mediterranean fruit fly, multiple experiments were performed using different restriction enzymes.
Randomization	No randomization was necessary as there were no comparisons between groups in this methods study.

Blinding	No blinding was necessary as there were no manual measurements in this methods study.

Reporting for specific materials, systems and methods

We require information from authors about some types of materials, experimental systems and methods used in many studies. Here, indicate whether each material, system or method listed is relevant to your study. If you are not sure if a list item applies to your research, read the appropriate section before selecting a response.

Materials & experimental systems

n/a	Involved in the study
☒	☐ Antibodies
☐	☒ Eukaryotic cell lines
☒	☐ Palaeontology and archaeology
☐	☒ Animals and other organisms
☒	☐ Clinical data
☒	☐ Dual use research of concern
☒	☐ Plants

Methods

n/a	Involved in the study
☒	☐ ChIP-seq
☒	☐ Flow cytometry
☒	☐ MRI-based neuroimaging

Eukaryotic cell lines

Policy information about cell lines and Sex and Gender in Research

Cell line source(s)	Human female LCL GM12878 was purchased from Coriell Institute for Medical Research.
Authentication	HiFi sequence data produced from GM12878 data was compared with existing Illumina data to authenticate materials. Cell identity was verified by expected genomic profiles from sequencing. GM12878 was also provider authenticated.
Mycoplasma contamination	Mycoplasma contamination was tested using PCR detection kit for the GM12878 cell line.
Commonly misidentified lines (See ICLAC register)	*Name any commonly misidentified cell lines used in the study and provide a rationale for their use.*

Animals and other research organisms

Policy information about studies involving animals; ARRIVE guidelines recommended for reporting animal research, and Sex and Gender in Research

Laboratory animals	Ceratitis capitata, strain CDFA-Waimanalo (California Department of Food and Agriculture), adult male, 1 used in study; Anopheles coluzzii, strain Ngousso , adult male, 1 used in study.
Wild animals	None.
Reporting on sex	In order to capture both sex chromosomes and a known translocation, a male C. capitata was chosen for the study.
Field-collected samples	None.
Ethics oversight	None required for insects.

Note that full information on the approval of the study protocol must also be provided in the manuscript.

Plants

Seed stocks	*Report on the source of all seed stocks or other plant material used. If applicable, state the seed stock centre and catalogue number. If plant specimens were collected from the field, describe the collection location, date and sampling procedures.*
Novel plant genotypes	*Describe the methods by which all novel plant genotypes were produced. This includes those generated by transgenic approaches, gene editing, chemical/radiation-based mutagenesis and hybridization. For transgenic lines, describe the transformation method, the number of independent lines analyzed and the generation upon which experiments were performed. For gene-edited lines, describe the editor used, the endogenous sequence targeted for editing, the targeting guide RNA sequence (if applicable) and how the editor was applied.*
Authentication	*Describe any authentication procedures for each seed stock used or novel genotype generated. Describe any experiments used to assess the effect of a mutation and, where applicable, how potential secondary effects (e.g. second site T-DNA insertions, mosiacism, off-target gene editing) were examined.*
